# Stochastic Model of *Tsc1* Lesions in Mouse Brain

**DOI:** 10.1371/journal.pone.0064224

**Published:** 2013-05-16

**Authors:** Shilpa Prabhakar, June Goto, Xuan Zuang, Miguel Sena-Esteves, Roderick Bronson, Jillian Brockmann, Davide Gianni, Gregory R. Wojtkiewicz, John W. Chen, Anat Stemmer-Rachamimov, David J. Kwiatkowski, Xandra O. Breakefield

**Affiliations:** 1 Molecular Neurogenetics Unit, Department of Neurology and Center for Molecular Imaging Research, Department of Radiology, Massachusetts General Hospital, and Program in Neuroscience, Medical School, Boston, Massachusetts, United States of America; 2 Translational Medicine Division, Department of Medicine, Brigham and Women’s Hospital, Harvard Medical School, Boston, Massachusetts, United States of America; 3 Neurology Department, Gene Therapy Center, University of Massachusetts Medical School, Worcester, Massachusetts, United States of America; 4 Rodent Histopathology Core Facility, Harvard Medical School, Boston, Massachusetts, United States of America; 5 Department of Pathology, Massachusetts General Hospital, Boston, Massachusetts, United States of America; 6 Center for Systems Biology and Department of Radiology, Massachusetts General Hospital, Boston, Massachusetts, United States of America; University of Kansas Medical Center, United States of America

## Abstract

Tuberous sclerosis complex (TSC) is an autosomal dominant disorder due to mutations in either TSC1 or TSC2 that affects many organs with hamartomas and tumors. TSC-associated brain lesions include subependymal nodules, subependymal giant cell astrocytomas and tubers. Neurologic manifestations in TSC comprise a high frequency of mental retardation and developmental disorders including autism, as well as epilepsy. Here, we describe a new mouse model of TSC brain lesions in which complete loss of *Tsc1* is achieved in multiple brain cell types in a stochastic pattern. Injection of an adeno-associated virus vector encoding Cre recombinase into the cerebral ventricles of mice homozygous for a *Tsc1* conditional allele on the day of birth led to reduced survival, and pathologic findings of enlarged neurons, cortical heterotopias, subependymal nodules, and hydrocephalus. The severity of clinical and pathologic findings as well as survival was shown to be dependent upon the dose and serotype of Cre virus injected. Although several other models of TSC brain disease exist, this model is unique in that the pathology reflects a variety of TSC-associated lesions involving different numbers and types of cells. This model provides a valuable and unique addition for therapeutic assessment.

## Introduction

Tuberous sclerosis complex (TSC) is a genetic disorder affecting about 1 in 6,000 newborns caused by inactivating mutations in *Tsc1*or *Tsc2,* encoding hamartin and tuberin, respectively [1], [2]. Biallelic loss of either gene leads to chronic hyperactivation of mTOR complex 1 (mTORC1), and this appears to be the primary pathogenetic mechanism that leads to development of TSC hamartomas in brain, kidney, skin, heart and lung [3], [4]. Focal brain pathologies, including cortical tubers and subependymal nodules (SENs), are seen in the majority (>90%) of TSC patients, and have been detected as early as late fetal gestation [5]. TSC tubers disrupt neuronal laminar architecture, and tuber size and number correlate with the incidence of infantile spasms and epileptic seizures [6], as well as global developmental delay [7]. Most TSC patients develop multiple neurological manifestations including seizures, intellectual deficit, neurobehavioral syndromes including autism and autism spectrum disorder, and sleep disorders [3]. Five to 10% of SENs show progressive enlargement, are then called subependymal giant cell astrocytomas (SEGAs), and can lead to devastating neurologic consequences due to blockage of cerebrospinal fluid (CSF) flow and progressive hydrocephalus.

Although there is clear evidence that loss of a single allele of *Tsc1*or *Tsc2* can affect global brain function [8], [9], both tuber giant cells and SEGA cells show evidence of complete loss of the TSC1/TSC2 complex with constitutive activation of mTORC1, augmented protein translation [10], reduced autophagy [11], [12], and endoplasmic reticulum (ER) and oxidative stress [13]. In addition, cortical tubers contain much higher levels of inflammatory cytokines than normal brain [14], suggesting an inflammatory contribution to TSC brain pathogenesis in humans.

A number of mouse models of TSC brain disease have been generated using conditional alleles of either *Tsc1* or *Tsc2*, and a variety of Cre recombinase alleles driven by different brain-specific promoters, typically active during embryonic development, and in some cases drug-inducible. Promoters have included those selective for neuroprogenitor cells, neurons and astrocytes (e.g. [9], [15]–[23]). In general widespread recombination in brain cells is seen in these models, inducing several features of TSC, such as epileptic seizures, prenatal onset of giant cell development, abnormal brain development (including heterotopias), decreased myelination, and hydrocephalus and premature death. In these conditional models, hamartin or tuberin loss occurs in essentially all of a specific subtype of brain cells at a particular time in development, in contrast to human patients where it occurs in a subset of different cell types at various times in development. More selective loss of *Tsc1* was achieved by *in utero* electroporation of a Cre recombinase expression cassette under a strong constitutive promoter into one hemisphere of embryonic conditional mice, which led to localized white matter heterotopic nodules and tuber-like lesions [24].Given the severity of neurological and cognitive deficits in TSC, efforts continue to try to develop mouse models which recapitulate TSC brain lesions as closely as possible in order to understand the pathophysiology and explore treatment paradigms.

In this study we aimed to recreate the stochastic nature of *Tsc1* loss of function in human brains with respect to subsets of different cell types being affected in order to model TSC brain pathology and its effects. Loss of *Tsc1* was induced by intracerebral ventricular (ICV) neonatal injection of adeno-associated virus (AAV) vectors encoding Cre recombinase, or green fluorescent protein (GFP) as a control, under a strong constitutive promoter. Two serotypes of AAV - AAVrh8 and AAV1 were used (the latter at two titers), each expressing Cre driven by a strong constitutive promoter, which has been shown to transduce a variety of cell types throughout the mouse brain following ICV injections at birth (P0) [25]. AAV-Cre vectors were injected ICV into *Tsc1^c/c^*ROSA pups at P0. AAV-Cre injected mice died prematurely with varying degrees of *lacZ+* staining and brain pathology, including enlarged brains with an abnormally smooth surface and hydrocephalus. Immunocytochemical staining revealed scattered enlarged neurons in the cortex and small cortical clusters of cells with dual differentiation shown by immunostaining for both neuronal and glial markers, as well as high phospho-S6 (pS6, Ser235/236) expression in astrocytes and other cells indicative of *Tsc1* loss and mTORC1 hyperactivation. Thickening of the subependymal layer of the ventricles was also noted, in some cases with small nodules in the adjacent intraventricular cerebrospinal fluid (CSF). These nodules stained positively for *lacZ*, doublecortin (DCX), glial fibrillary acidic protein (GFAP) and the transmembrane glycoprotein (GPNMB), similar to SENs seen in TSC patients.

## Methods

### AAV Vector Design and Packaging

AAV vector plasmid, AAV-CBA-Cre-BGHpA was derived from the plasmid AAV-CBA-EGFP-W [25] by replacing EGFP and WPRE element with the Cre recombinase cDNA. The AAV-CBA-EGFP-W vector was used as a control. These AAV vectors carry AAV2 ITR elements and gene expression is controlled by a hybrid promoter (CBA) composed of the cytomegalovirus (CMV) immediate/early gene enhancer fused to the chicken beta-actin promoter. The identity of all PCR amplified sequences was confirmed by sequencing.

AAVrh8 and AAV1 serotype vectors were produced by transient co-transfection of 293T cells by calcium phosphate precipitation of vector plasmids (AAV-CBA-Cre or AAV-CBA-GFP-W), adenoviral helper plasmid pFΔ6 and a plasmid encoding for the AAVrh8 cap (pAR-rh8) or AAV1 cap gene (pXR1), as previously described [25]. Briefly, AAV vectors were purified by iodixanol gradient centrifugation followed by column chromatography using HiTrapQ anion exchange columns (GE Healthcare, Piscataway, NJ, USA). The virus-containing fractions were concentrated using Centricon 100 kDa MWCO centrifugal devices (EMD Millipore, Billerica, MA, USA) and the titer [genome copies (g.c.)/ml] was determined by real-time PCR amplification with primers and probe specific for the bovine growth hormone polyadenylation signal.

### Animals and ICV Injections

Experimental research protocols were approved by the Institutional Animal Care and Use Committee (IACUC) for the Massachusetts General Hospital (MGH) following the guidelines of the National Institutes of Health for the Care and Use of Laboratory Animals. Experiments were performed on *Tsc1^c/c^* mice which also carried the Cre-inducible ROSA26 *lacZ* marker allele, as described [26], [27]. In response to Cre recombinase the *Tsc1^c/c^* allele is converted to a null allele, and the *lacZ* allele expresses β-galactosidase. These mice have a normal lifespan.

For vector injections, on the day of birth (P0), neonates were cryo-anesthetized and injected with 2 µl of viral vector into each cerebral lateral ventricle with a glass micropipette (70–100 µm diameter at the tip) using a Narishige IM300 microinjector at a rate of 2.4 psi/sec (Narshige International, East Meadow, NY, USA). The viral vector solution consisted of either 2×10^10^ g.c. per 2 µl or 2×10^9^ g.c. per 2 µl. Mice were then placed on a warming pad and returned to their mothers after regaining normal color and full activity typical of newborn mice. Mice were euthanized when they showed a weight loss of >15%, greatly reduced movement or other signs of distress.

### X-gal Staining

Mice were sacrificed using CO_2_ generated from dry ice and brains were rapidly frozen in 2-methyl-butane/dry ice bath. Coronal serial sections were cut to a thickness of 10 µm using a cryostat, directly mounted on glass slides and stored at −80°C. Slides were fixed in 0.25% glutaraldehyde in PBS (pH 7.4) for 20 min, stained in X-gal solution (Gold Biotechnology, St. Louis, MO, USA) overnight at 37°C and counterstained with Nuclear Fast Red solution (Vector Laboratories, Burlingame, CA, USA).

### Histology and Immunohistochemistry (IHC)

For standard histologymouse brains were prepared after euthanasia with CO_2_by immediate removal of brains and 2–4 days of fixation in Bouin’s solution (VWR International, Radnor, PA). Following paraffin embedding, 5 µm sections were cut and stained with either Haematoxylin and Eosin (H&E) or were used for IHC. IHC was performed after deparaffinization and re-hydration steps and antigen retrieval in citrate buffer (pH 6) using the EnVision System (Dako, Carpinteria, CA, USA) or HistoMouse-Plus kit (Invitrogen, Carlsbad, CA, USA), per manufacturer’s instruction. pS6 antibody (#2211) and TSC2 antibody (#4308) were from Cell Signaling (Danvers, MA, USA).

For DCX, GFAP, NeuN and GPNMB staining, sections were deparaffinized in xylenes followed by re-hydration in decreasing ethanol concentrations. Endogenous peroxidase was blocked with 0.5% hydrogen peroxide, then tissues were washed in PBS. Heat-induced antigen retrieval was performed using sodium citrate 10 mM, pH 6.0, in a 95°C water bath (20 min for GPNMB and 30 min for DCX). Tissues were blocked in a 10% normal goat serum (GPNMB) or 10% normal horse serum (GFAP, DCX, NeuN) then incubated overnight with primary antibody. Dilutions were: 1∶300 rabbit polyclonal GPNMB (Lifespan Biosciences, Seattle, WA, USA) LS-C80662/28556, 1∶250 goat polyclonal DCX (Santa Cruz Biotechnologies, Santa Cruz, CA, USA, sc-8066), 1∶400 mouse monoclonal NeuN (MAB377, EMD Millipore) and 1∶800 mouse monoclonal GFAP (Sigma, St. Louis, MO, USA, C9205). Tissues were incubated for 30 min with secondary antibody 1∶250 biotinylated goat anti-rabbit (Vector Laboratories, BA-1000), 1∶250 biotinylated horse anti-goat (Vector Laboratories, BA-9500) and 1∶250 and 1∶400 biotinylated horse anti-mouse (Vector Laboratories, BA-2000), washed in PBS and incubated with avidin-biotin complex (Vector Laboratories, PK-6100) for 30 min. Staining was performed with 3,3′-diaminobenzidine (DAB) in H_2_O_2_ (Vector Laboratories), then counterstained with haematoxylin, dehydrated and coverslipped.

### Immunostaining with NeuN, GFAP and pS6; Neuronal Cell Measurements

Mice were sacrificed at 1 month of age by transcardiac perfusion with PBS followed by ice-cold 4% paraformaldehyde in PBS. Brains were dissected and post-fixed for 4 hrs at 4°C, followed by overnight incubation in 30% sucrose in PBS at 4°C and were embedded in tissue freezing medium (Tissue-Tek O.C.T compound, Sakura Finetek Inc., Torrance, CA, USA). Coronal serial sections were cut to a thickness of 10 µm and directly mounted on glass slides. Sections were stained for the neuronal marker, 1∶1000 mouse monoclonal NeuN (MAB377, EMD Millipore) or glial marker, 1∶500 mouse monoclonal anti-GFAP (Clone G-A-5 Cy3 conjugate, Sigma), or for pS6 - 1∶1000 pS6 rabbit antibody (Cell Signaling) in 0.1% Tween-20 in PBS overnight at 4°C, washed in PBS 3×10 min, incubated for 30 min with 1∶1000 Alexa 488-conjugated goat anti-mouse secondary antibody (Life Technologies, Grand Island, NY, USA) or 1∶1000 Alexa 546-conjugated anti-rabbit secondary antibody (Life Technologies) in 0.1% Tween-20 in PBS. After another 3×10 min washes in PBS, sections were counterstained with 4,6 diamidino-2-phenylindole (DAPI, Sigma) for 5 min, washed in PBS and coverslipped.

In NeuN immunofluorescence stained brains, the widest diameter of NeuN+ cells in cortex was measured using Metavue software (Molecular Devices, Sunnyvale, CA, USA) for 180 randomly selected cells in the cortex just above the lateral ventricles from 3 animals in each group. In NeuN immunostained brain, the widest diameter of NeuN+ cells in cortex was measured using photoshop software (Adobe) for 30 randomly selected cells in the cortex just above the lateral ventricles from 3 animals in each group.

### MRI

The mice were scanned using a T2-weighted TurboRARE-3D (TE = 43 ms and TR = 1200 ms) sequence for a 192×256×192 matrix and a voxel size of 0.0976×0.130×1.302 mm after reconstruction on a Bruker Pharmascan 4.7 tesla magnet using a Bruker mouse brain coil (Model T8118, Bruker Scientific Instruments, Billerica, MA USA). Regions of interest (ROI) of the brains were automatically segmented using a house-built Matlab program by searching for the largest connected region of a given threshold. These ROI’s were manually modified using Amira software (Amira, Burlington, MA, USA) to check for any anomalies in the auto-segment routine. An additional ROI was manually drawn to represent the normal brain tissue and, utilizing a region-based threshold that 3 standard deviations above the mean normal brain value, the CSF was segmented. This segmented region was manually modified by removing any spurious voxels above this threshold around the edges of the brain that were due to partial volume effect.

### Statistical Analysis

All analyses of survival curves (Chi square test) and brain ventricular volumes (t test) were performed using GraphPad Prism software (GraphPad Software, Inc., La Jolla, CA, USA). The p values depicted are statistically significant.

## Results

### AAVrh8-CBA-Cre Injections into *Tsc1^c/c^* Mice

**Figure 1:**
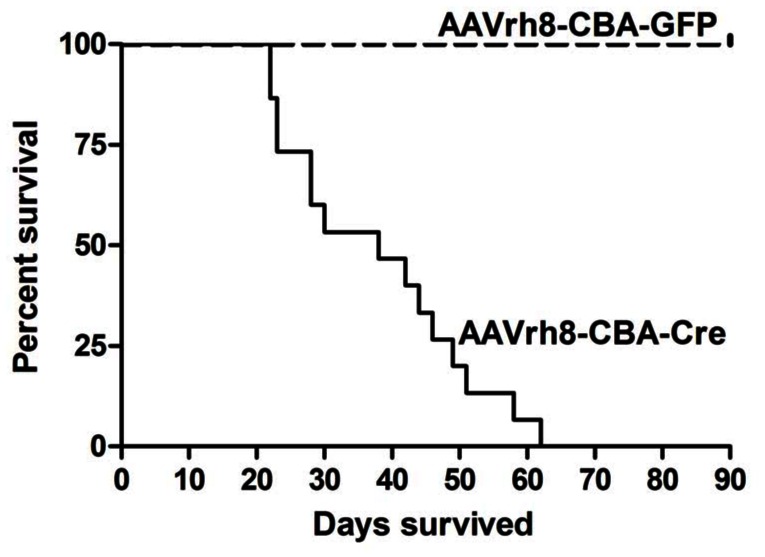
Survival of *Tsc1^c/c^* mice injected ICV with AAVrh8-CBA-GFP or AAVrh8-CBA-GFP at P0. Survival is shown for *Tsc1^c/c^* pups injected with the AAV-Cre (N = 15) or the AAV-GFP (N = 13) vectors as a control, both at 2×10^10^ g.c. per 2 µl into each ventricle. Median survival of the Cre injected mice was 38 days; controls survived >90 days. Difference between groups p<0.0001.

We initially evaluated the survival of *Tsc1^c/c^* mice receiving an ICV injection of AAVrh8-CBA-Crevectorat P0 (2×10^10^ g.c. per 2 µl into each ventricle). In contrast to mice receiving a control injection of an AAVrh8-CBA-GFP vector, AAVrh8-CBA-Cre injected mice showed a median survival of 38 days (**Fig. 1**, p<0.0001). GFP immunostaining of brains of AAVrh8-CBA-GFP injected mice and non-injected mice at 110 days showed GFP positive cells of different morphologies throughout the periventricular zone and cortex of the former, with no staining in the latter (**Fig. S1**).

**Figure 2:**
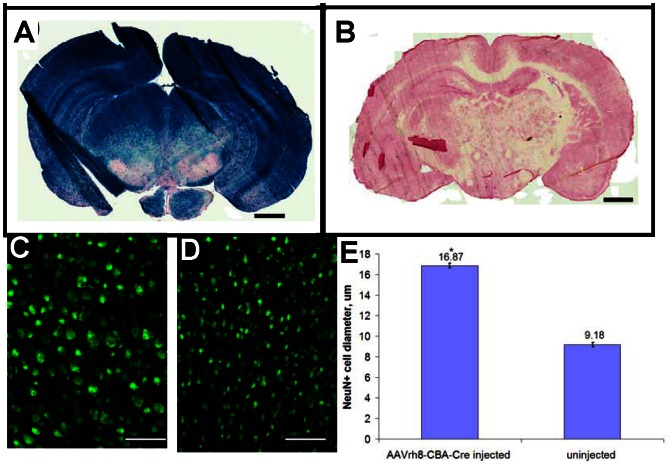
Cre-mediated recombination in *Tsc1^c/c^* mice after injection of AAVrh8-CBA-Cre at birth. In *Tsc1^c/c^* mice, Cre-mediated recombination knocks-out the floxed *Tsc1* allele and knocks-on a transgenic *lacZ*gene. These mice were injected at P0 with AAV8-CBA-Cre vector (2×10^10^g.c. in each ventricle), and *lacZ* expression throughout the brain was analyzed at ∼P28 as a marker of recombination efficiency. (A) AAV-treated *Tsc1^c/c^*ROSA mice displayed intense *lacZ* expression throughout the brain, (B) while uninjected controls were devoid of any *lacZ* activity. Ten µm sections were stained with X-gal solution and counterstained with Nuclear Fast Red. Scale bars: 1 mm. (C & D) Staining for NeuN shows that neurons above the lateral ventricles in AAVrh8-CBA-Cre injected mice were considerably larger than in control uninjected mice Scale bars: 200 µm. (E) The periventricular neurons of the brains of AAV-Cre injected and non-injected mice (P28) were immunostained for the neuronal marker, NeuN and the widest diameter of stained cell bodies was measured in >180 randomly selected cells from several fields with 3 animals per group. The AAV-Cre injected cortical neurons had an almost 2-fold increase in diameter, shown as mean±SEM (*p<0.001).

Brains obtained from *Tsc1^c/c^* mice injected with the AAVrh8-CBA-Cre vector collected at P30 (N = 3) appeared enlarged and swollen. Staining with *lacZ* typically showed widespread evidence of recombination (**Fig. 2A**), while brains from non-injected *Tsc1^c/c^* mouse controls at the same age showed no *lacZ* staining (**Fig. 2B**). NeuN staining (**Fig. 2C and D**) demonstrated that neurons near the periventricular area chosen from each brain had an average diameter of 16.9±0.22 µm in AAVrh8-CBA-Cre injected mice in comparison to 9.2±0.21 µm for non-injected animals (**Fig. 2E**), representing an almost 2-fold increase in neuronal diameter in the *Tsc1* knock-out brains (p<0.001).

**Figure 3:**
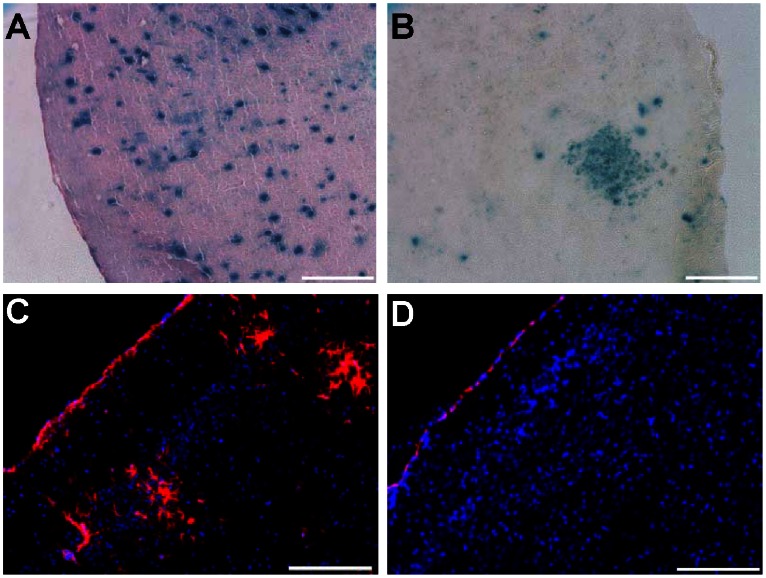
*LacZ* and GFAP expression in AAVrh8-CBACre injected *Tsc1^c/c^*ROSA mice. *Tsc1^c/c^*ROSA mice were injected at P0 with AAVrh8-CBA-Cre vector (2×10^9^g.c. in each ventricle), and *lacZ* expression in the brain was analyzed at 6 months (N = 2). (A & B) *LacZ*+ cells were observed scattered as single cells or in patches throughout the cortex. (C & D) Immunocytochemical staining for GFAP with DAPI dye staining of nuclei revealed numerous foci of GFAP+ (red) cells in the cortex (C) while uninjected controls were devoid of any GFAP+ cells in the cortex (D). Scale bars: A–D: 200 µm.

**Figure 4:**
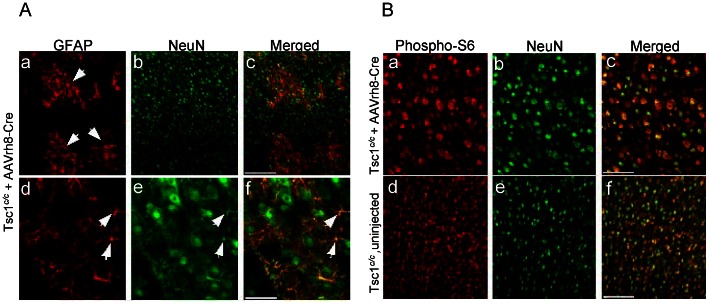
GFAP, NeuN and pS6 staining in brains of AAVrh8-CBA-Cre injected and non-injected *Tsc1^c/c^*ROSA mice at 1 month. (A) Co-immunostaining for GFAP and NeuN in AAVrh8-CBA-Cre injected *Tsc1^c/c^*ROSA mice showed clusters of GFAP+ cells in the cortex, some of which co-stained for NeuN (arrows). Scale bars: a–c-:200 µm; d–f: 50 µm. (B) Co-immunostaining for pS6 and NeuN in AAVrh8-CBA-Cre injected *Tsc1^c/c^*ROSA mice and non-injected mice (a–c) Cells were larger in AAVrh8-CBA-Cre injected mice than in controls and stained more strongly for pS6. Scale bars: a–f: 200 µm.

Since the above mice demonstrated high level and global brain recombination, we also injected two *Tsc1^c/c^* mice with a lower dose of AAVrh8-CBA-Cre 2×10^9^ g.c. per 2 µl into each ventricle (1/10^th^ that of above animals). These two mice survived for 180 days when they developed mild signs of distress characterized by a hunched back. Neuropathologic examination of their brains showed fewer *lacZ*+ cells, occurring in a scattered pattern and in clusters throughout the cortex (**Fig. 3A and B**). Staining for an astrocytic marker (GFAP) in these mice revealed clusters of positive cells in the AAVrh8-CBA-Cre-injected *Tsc1^c/c^* mice (**Fig. 3C**), which were absent in un-injected *Tsc1^c/c^* mouse brain (**Fig. 3D**). Some of the cells in GFAP+ clusters in AAVrh8-CBA-Cre-injected *Tsc1^c/c^*ROSA pups also stained for NeuN suggesting a possible mixed cell lineage phenotype (**Fig. 4A**), although the small size of the apparently co-staining cells was unexpected. Further, enlarged neurons in AAVrh8-CBA-Cre-injected *Tsc1^c/c^* mice showed strong staining for pS6 (**Fig. 4B**).

**Figure 5:**
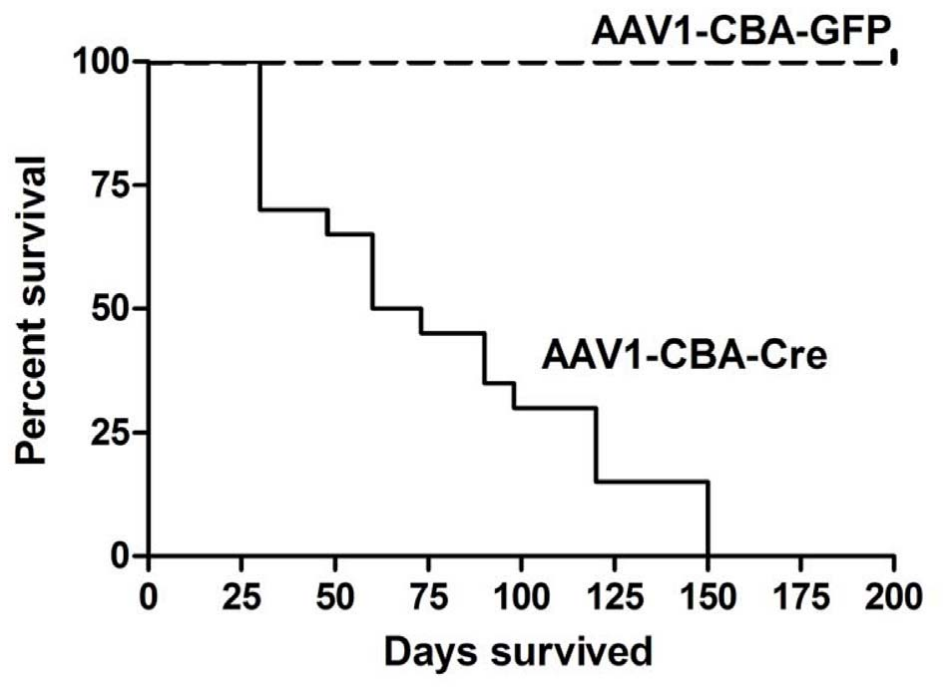
Survival data (Kaplan-Meier curve) of *Tsc1^c/c^*ROSA mice injected ICV with AAV1-CBA-GFP or AAV1-CBA-Cre at P0. Death was determined by the point at which animals were in severe distress at which time they were euthanized. *Tsc1^c/c^*ROSApups (N = 20) injected with the AAV-CBA-Cre vector died over a range of 30–150 day postnatal period, with a mean of 66.5 survival days. The same P0 pups injected ICV with AAV-GFP as a control (dashed line) all survived >200 days, the longest time point analyzed (N = 8). p<0.0001.

To achieve a milder phenotype than in the *Tsc1^c/c^* mice injected with high titer AAVrh8-CBA-Cre, we also tested an AAV1-CBA-Cre vector since AAV1 vectors have been shown to transduce fewer brain cells in mouse pups after neonatal ICV injection [25]. ICV injection of AAV1-CBA-Cre vector (2×10^10^ g.c. per 2 µl into each ventricle) in P0 *Tsc1^c/c^* pups produced a somewhat longer median survival time (median of 66.5 days, range 30–150 days), while parallel injection of an AAV1-CBA-GFP vector did not result in any animal death over 200 days (**Fig. 5**, p<0.0001). To confirm that recombination had occurred at the *Tsc1* locus in the brains of mice subject to injection of the AAV1-CBA-Cre, we performed multiplex ligation-dependent probe assay (MLPA). MLPA can be used to determine the extent of recombination of the c to the k (null) allele at *Tsc1* in a quantitative fashion, as described [28], [29]. Ten % recombination was found in the brains of AAV1-CBA-Cre injected animals and 0% in AAV1-CBA-GFP injected animals (**Fig. S2**).

Extensive histological analysis was carried out on *Tsc1^c/c^* mice following P0 ICV injection of AAV1-CBA-Cre or AAV1-CBA-GFP sacrificed at different time points. Severe hydrocephalus was seen in 2 of 10 AAV1-CBA-Cre injected brains, and mild hydrocephalus in 6 of 10 brains in animals sacrificed at 1–5 months of age due to signs of distress, including hunched back, dehydration and weight loss. In contrast, mild hydrocephalus was seen in only 1in 6 brains of animals injected with the AAV1-CBA-GFP control vector (p<0.0041; **Table S1**) and none of the control animals showed signs of distress, nor did any of the AAV1-CBA-GFP injected controls in the survival curves out to 200 days (**Fig. 5**).

**Figure 6:**
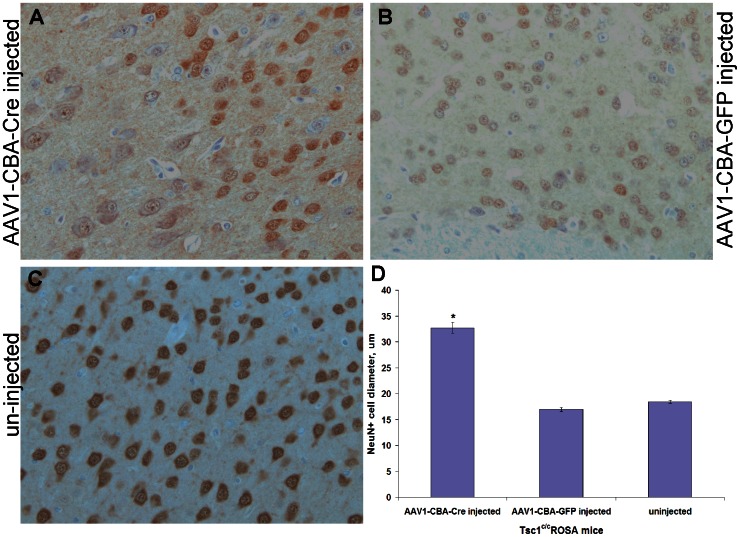
NeuN staining for neuronal diameter measurements. Immunostaining for NeuN (counterstained with haematoxylin) shows that neurons above the lateral ventricles in (A) AAV1-CBA-Cre injected *Tsc1^c/c^* mice were considerably larger than those in controls in the same region: (B) AAV1-CBA-GFP injected and (C) uninjected mice. Magnification = 40X. (D) Periventricular neurons in the brains of AAV1-Cre and AAV1-GFP injected, as well as non-injected mice (P30) were stained for the neuronal marker, NeuN and the widest diameter of stained cell bodies was measured in >30 randomly selected cells from several fields with 3 animals per group. Neurons in the AAV1-CBA-Cre injected brains had an almost 2-fold increase in diameter, as compared to controls, shown as mean±SEM (*p<0.0001).

Brains obtained from *Tsc1^c/c^* neonatal mice injected with the AAV1-CBA-Cre vector collected at P30 (N = 3) appeared enlarged, as compared with parallel mice injected with the AAV1-CBA-GFP vector or non-injected (N = 3, respectively; data not shown). NeuN staining in AAV1-CBA-Cre injected brains (**Fig. 6A**) demonstrated that neurons near the periventricular region had an average diameter of 32.7±6.0 µm, in comparison to 17.0±2.4 µm for AAV1-CBA-GFP injected mice (**Fig. 6B**) and 18.4±2.6 µm for non-injected animals (**Fig. 6C**), representing an almost 2-fold increase in neuronal diameter in the *Tsc1* knock-out brains (**Fig. 6D**; p<0.0001).

**Figure 7:**
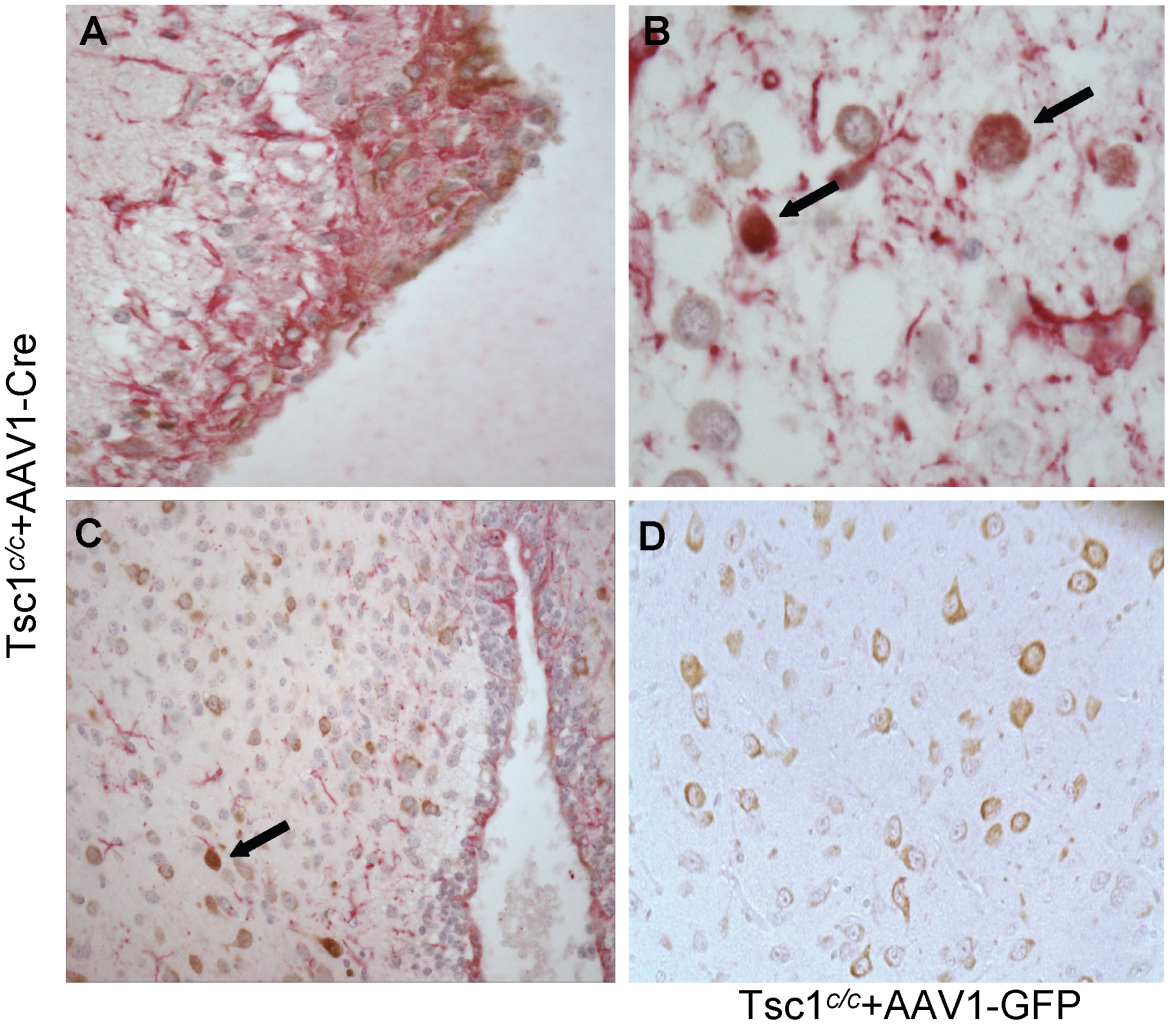
pS6 and GFAP double immunostaining to assess astrocyte transduction. *Tsc1^c/c^*ROSA homozygous pups were injected ICV at P0 with either an AAV1-CBA-GFP or AAV1-CBA-Cre vector at a concentration of 2×10^10^g.c. One month later two of the pups injected with AAV1-CBA-Cre virus who developed distress were sacrificed and showed severe hydrocephalus by neuropathological examination. To assess astrocyte transduction, the brains were double stained for pS6 and GFAP and counter stained with haematoxylin. Intermediate or mixed cells which stain for both the markers were seen in the (A) periventricular region and (B) subependymal lining. No co-localization was seen in the cortex except for a very few cells(C). No double staining was seen in any region of the brain in AAV1-GFP injected brain (D). Arrows indicate double stained cells. Magnification = 40X.

**Figure 8:**
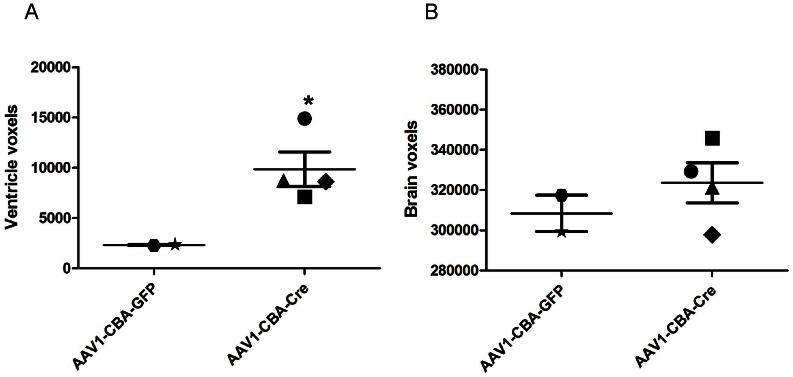
Quantitative volumetric analysis of MR images of *Tsc1^c/c^* pups at 1 month of age after P0 ICV injection. Injection of AAV1-CBA-Cre (N = 4) or AAV1-CBA-GFP (N = 2) 2×10^10^ g.c. per 2 µl into each ventricle was carried out on P0 and MR images were evaluated on P30. (A) Measurement of ventricle size in voxels (each voxel = 0.0976×0.130×1.302 mm).Difference between groups is significant (p<0.044). (B) Measurement of brain parenchyma (excluding ventricle volume) in voxels. Difference between groups, p<0.23, not significant. Measurements were made by observer blinded to genotype.

**Figure 9:**
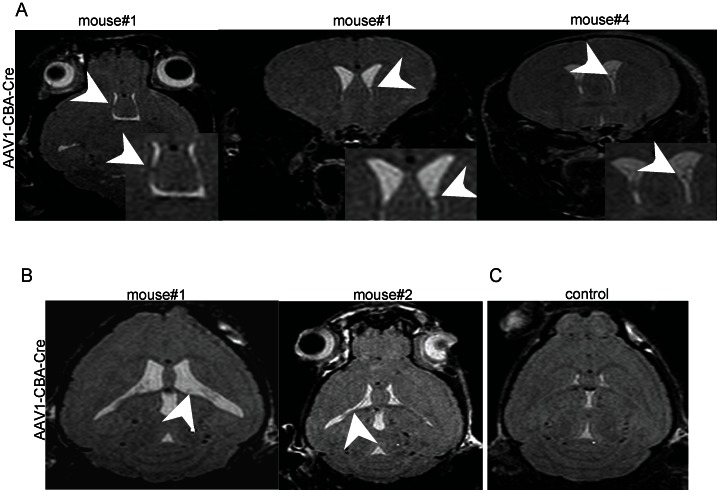
Representative MR images of AAV1-CBA-Cre and AAV1-CBA-GFP injected mouse brains. AAV1-CBA-Cre injected mouse brains (see Fig. 8 legend) showed abnormalities associated with the ventricular regions. (A) Multiple apparent subependymal nodules (arrowheads) were seen in the ventricles in the brains of two AAV1-CBA-Cre injected mice. First two images are from the same mouse. (B) Ventricles also appeared to have thickening of the ependymal lining (arrowheads; two left panels). (C) None of these abnormalities were observed in the control vector (AAV1-CBA-GFP)-injected brains.

To assess astrocyte transduction, brains of AAV1-CBA-Cre and AAV1-CBA-GFP neonatal injected *Tsc1^c/c^* mouse brains at P30 were co-stained with pS6 and GFAP antibodies and counterstained with haematoxylin. In the AAV1-CBA-Cre injected brains, intermediate or mixed cells stained for both the markers in the periventricular regions (**Fig. 7A**) and subependymal lining (**Fig. 7B**), which corresponds to the location of SEGAs in Tsc patients. Co-localization in the cortex was seen only in very few cells (**Fig. 7C**). No co-staining was seen in the AAV1-CBA-GFP injected brains (**Fig. 7D**). Pairs of AAV1-CBA-Cre and AAV1-CBA-GFP injected *Tsc1^c/c^* mouse brains were examined at P30, demonstrating that brains of AAV1-CBA-Cre-injected mice were enlarged and had a smoother surface, as compared to control vector injected brains, although the weight of the brains was not significantly different between the two groups (data not shown). Four mice injected ICV at P0 with AAV1-CBA-Cre and 2 mice injected in parallel with AAV1-CBA-GFP were examined by *in vivo* magnetic resonance imaging (MRI) [4.7 Tesla (T)] at P30 with volumetric analysis of brain regions. Ventricular volume was about 4 times larger in the AAV1-CBA-Cre injected vs. control injected mice (**Fig. 8A**, p<0.044), while the brain tissue volume was only about 6% larger on average in the former (**Fig. 8B**, p value non-significant). In addition to enlarged ventricles, in three of the four AAV1-CBA-Cre injected mice, nodules and thickening of the ventricular lining were noted (arrowheads in **Fig. 9A and B**), while no abnormalities were seen in control vector injected mice (**Fig. 9C**). In one AAV1-CBA-Cre injected mouse brain there was an apparent increased geographic signal abnormalities in the brain, which may represent cortical tubers or dysplasia, not seen in controls (**Fig. S3**).

**Figure 10:**
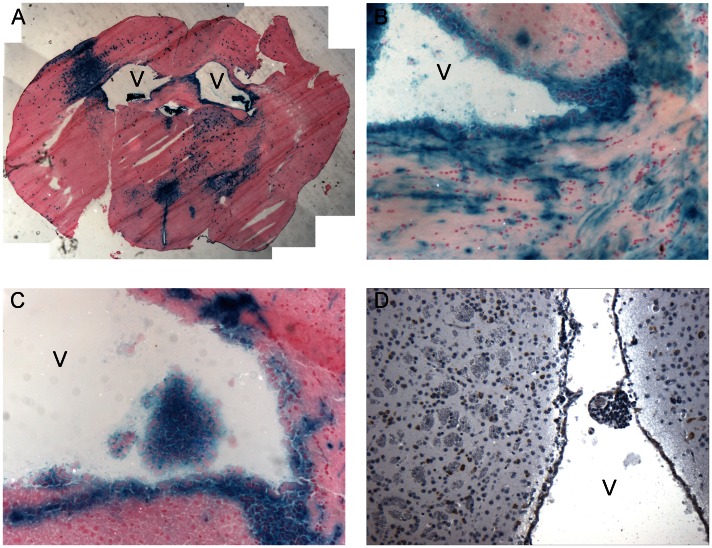
Cre-mediated recombination in *Tsc1^c/c^* mice after neonatal delivery of AAV1-CBA-Cre vector (2×10^10^ g.c. per 2 µl into each ventricle). (A) Three months after ICV vector injection into *Tsc1^c/c^*ROSA homozygous pups, frozen sections were stained with X-gal and counterstained with Nuclear Fast Red. Intense *lacZ* staining was visualized in clusters throughout the brain(coronal section, anterior to hippocampus region); note enlarged ventricles (V). Magnification = 2X. (B) The ventricular lining showed patches of *lacZ* staining and thickening. (C) Nodule-like structure, composed of cells in which Cre recombination has been induced as visualized by X-gal staining, were observed in the ventricles. (D) H&E staining showing nodule along ventricular lining. The regions in B, C & D are taken at a level close to the anterior amygdala. Magnification B, C & D = 10X.

*LacZ* staining of the brains of the AAV1-CBA-Cre injected *Tsc1^c/c^* mice sacrificed at 1 month revealed clusters of positive cells scattered throughout the brain and strong staining around the ventricles (**Fig. 10A**; *Note,* also the enlarged lateral ventricles.) Higher magnification of the ventricular regions showed thickening of the ventricular layer associated with *lacZ*+ cells indicative of recombination and *Tsc1* loss (**Fig. 10B**), as well as clusters of *lacZ*+ cells near the ventricles and a *lacZ+* nodule protruding into the ventricle (**Fig. 10C**), and a nodule of undifferentiated cells budding off the subependymal surface with clear staining of cell nuclei with haematoxylin (**Fig. 10D**).

**Figure 11:**
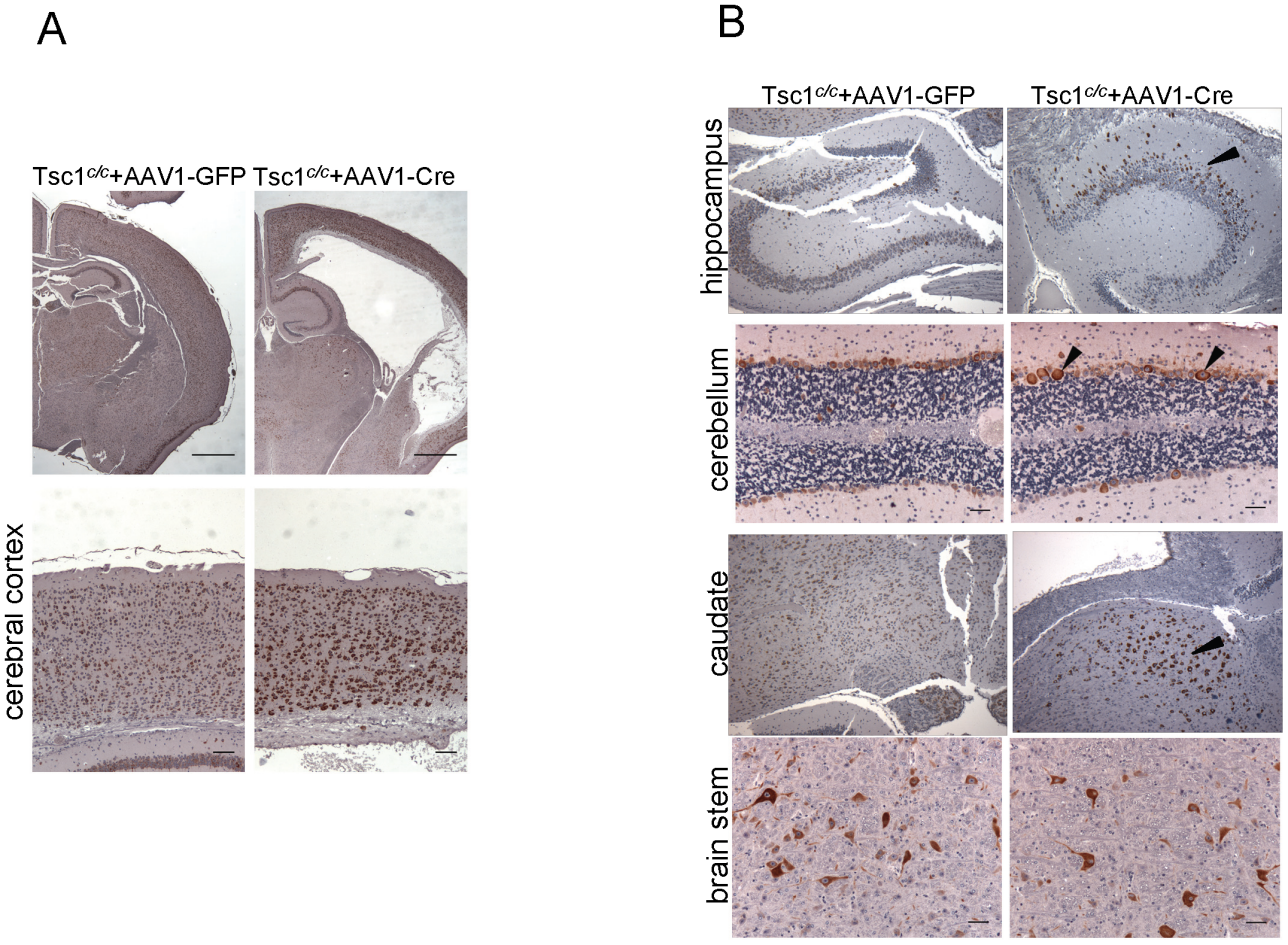
Hydrocephalus and enlarged pS6+ cortical cells in *Tsc1^c/c^* mice injected with AAV1-CBA-Cre vector. *Tsc1^c/c^*ROSA homozygous pups were injected ICV at P0 with either an AAV1-CBA-GFP (N = 6) or AAV1-CBA-Cre (N = 10) vector at a concentration of 2×10^10^g.c per ventricle. One month later pups which had received AAV1-CBA-Cre virus developed tremor (N = 8) and were found to have hydrocephalus by pathologic analysis (N = 2). (A) Representative images of brain (upper panels) and cerebral cortex (lower panels) showing in AAV1-CBA-Cre injected animals severe hydrocephalus, large portion of enlarged cortical cells with strong pS6-positivity, and condensed cortical thickness due to swelling of ventricles. Scale bars = 1 mm, upper; 100 µm, lower. (B) Representative pS6 staining in different brain regions. Enlarged pS6+ cells were ectopically present in striatum oriens of hippocampus (arrowhead) due to migration defect of *Tsc1*-null cells. Some pS6+ Purkinje cells in the cerebellum as well as neurons in the caudate were notably enlarged (arrowheads). Neural cells in deep nuclei such as the brain stem showed similar distribution of pS6 positivity in Cre and GFP injected mice. Scale bars = 50 µm.

Coronal sections of brains of AAV1-CBA-GFP and AAV1-CBA-Cre injected *Tsc1^c/c^* mice sacrificed at P30 showed massively enlarged lateral ventricles for the latter (**Fig. 11A**, top), which appeared to result from a constriction between the 3rd and lateral ventricles, as the 3rd ventricle, 4th ventricle and aqueduct appeared to be of normal size. Immunohistochemical staining for pS6 revealed strong signal in enlarged cells in the cortex of AAV1-CBA-Cre injected animals, as compared to AAV1-CBA-GFP injected animals (**Fig. 11A**, bottom). Enlarged pS6+ cells, as compared to controls, were seen throughout most regions of the brain of AAV1-CBA-Cre injected animals including in the hippocampus, cerebellum, and caudate, with less difference compared to controls seen in the brainstem (**Fig. 11B**).

**Figure 12:**
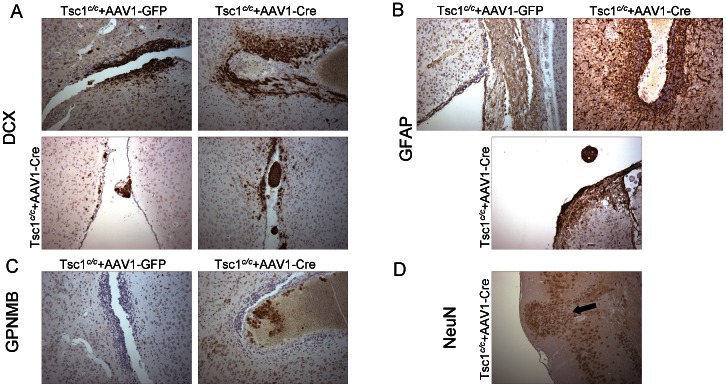
DCX, GFAP, GPNMB and NeuN staining in brains of AAV1-CBA-Cre and AAV1-CBA-GFP P0 injected *Tsc1^c/c^* mice at 1 month. *Tsc1^c/c^*ROSA homozygous pups were injected ICV at P0 with either an AAV1-CBA-GFP or AAV1-CBA-Cre vector at a concentration of 2×10^10^g.c. One month later pups injected with AAV1-CBA-Cre virus were sacrificed and processed for immunohistochemistry and showed severe hydrocephalus (N = 2). The pups (A) Staining for DCX revealed positive regions along the ventricles(anterior to the striatum, at the level of septal nuclei) in control animals (**Fig. 12A**, top left panel), but those regions were enlarged in AAV1-CBA-Cre injected animals (**Fig. 12A**, top right). In addition, the latter animals showed small DCX positive nodules [**Fig. 12A**, bottom left (level close to anterior amygdale) and right (level of septal nuclei)], which were not seen in control animals. (B) No strong GFAP staining were seen in the ventricular lining (level of septal nuclei) of control brains, but was intense in some subventricular regions (anterior to striatum) of the AAV1-CBA-Cre injected animals (**Fig. 12B**, top panels), including some GFAP positive nodules in the CSF space at the level of septal nuclei (**Fig. 12B**, bottom panel). (C) GPNMB staining was virtually null in control brains, with some positive cells near the ventricles anterior to striatum in AAV1-CBA-Cre injected animals (**Fig. 12C**). (D) NeuN staining of brains revealed rare heterotopias (arrow) consisting of outgrowth of glial cells into the cerebral cortex. Magnification = 20X.

In order to identify the phenotype of the abnormal ventricular structures in AAV1-CBA-Cre injected *Tsc1^c/c^* mice, as compared to AAV1-CBA-GFP injected animals, additional immunohistochemical staining was performed. Staining for doublecortin (DCX; a marker for migratory neuroblasts in the subventricular zone [30] revealed positive regions along the ventricles in control animals, but those regions were enlarged in AAV1-CBA-Cre injected animals (**Fig. 12A**, upper panels). In addition, the latter animals showed small DCX positive nodules attached to the ventricular wall or sometimes appearing to be floating in the CSF (**Fig. 12A**, lower panels), which were not seen in control animals. No strong GFAP staining was seen in the ventricular lining of control brains, but was intense in some subventricular regions of the AAV-CBA-Cre injected *Tsc1^c/c^* mice, including some GFAP positive nodules in the CSF space (**Fig. 12B**). We also noted strong staining for GPNMB (transmembrane glycoprotein found in human subependymal nodules) [31] in AAV1-CBA-Cre injected brains, but not in AAV1-CBA-GFP injected brains (**Fig. 12C**). In one AAV1-CBA-Cre injected animal immunostaining with NeuN highlighted a superficial cortical nodule formed by a focal herniation of cortical layers I and III through the molecular layer and neurons of various sizes grouped around a central blood vessel (**Fig. 12D**). Cortical nodules with a similar histological appearance in humans are known as nodular cortical dysplasia or “brain warts”. No other cortical lesions were observed at the neuropathological level in the four AAV1-CBA-Cre injected mice analyzed by MRI.

## Discussion

In this study we have generated a new model of TSC brain disease by exogenous injection of Cre expressing AAV virus at birth to pups that are homozygous for the conditional allele of *Tsc1, Tsc1^c/c^* mice. To our knowledge this method of induction of *Tsc1* or *Tsc2* loss in the developing brain has not been reported previously. Although many previous brain models of TSC have been described (for references, see Introduction), this new model has several features that are of interest. First, the brains of treated mice contain large numbers of pS6+ neurons reflecting effects of loss of *Tsc1* and development of cells that approach giant cells in their morphology. Second, at lower vector doses, we achieved variable, mosaic loss of *Tsc1* both in scattered cells and in focal regions, similar to cortical tubers. Third and arguably most interesting was the development of consistent hypertrophy of the subependymal layer, with expansion of the normal 1 cell thick layer into a convoluted layer with projections and apparently isolated nodules in the CSF. This subependymal proliferation appeared to be the cause of hydrocephalus in these mice, which was a major contributor to their morbidity and mortality.

Subependymal nodules (SENs) are seen in the great majority of TSC children. The potential for growth of these lesions has led to formal guidelines that recommend frequent periodic screening by MRI in TSC children and young adults. Further, a recent report indicated that about one third of SENs were observed to grow over a 4-year period postnatally [32]. Moreover, progressive increase in size of a SEN to a diameter >1 cm is seen in 5–10% of all children with TSC, necessitating intervention by either treatment with mTOR inhibitors, rapamycin [33] or everolimus [34], [35], or surgical removal [36], [37]. The consistent development of similar subependymal proliferative lesions in this new mouse model enables investigation of therapeutic strategies, including other mTOR inhibitors and novel approaches. The ability to titrate the dose of virus injected and thereby the severity of disease induced makes this model particularly useful in this regard.

Although rapamycin and related drugs represent a major breakthrough in the therapy of TSC tumors that develop at multiple sites [38], [39], there is continuing need for improvement in therapies, particularly those that address the diverse array of neurologic and neurobehavioral consequences of TSC brain involvement. Our current studies provide proof of concept that AAV vector delivery to the neonatal mouse brain can be exploited to induce a TSC phenotype. In future studies we hope to use this same approach to deliver *Tsc1* or *Tsc2* (rather than Cre recombinase) by similar means to brain cells, and reverse the complex set of neuropathology and phenotypes induced by loss of these genes. Hence, these studies are the first step in the exploration of possible gene therapy as a therapeutic strategy in TSC.

## Supporting Information

Figure S1 **GFP staining on the AAVrh8-CBA-GFP injected brains.** Brains of AAVrh8-CBA-GFP injected at P0 and uninjected *Tsc1^c/c^* mice at P30 at 110 days were stained for GFP and counter stained with haematoxylin. Positive staining was revealed throughout the brain, shown here in the cortex in the cortex and the ventricles in the AAV injected mice. Magnification = 20X.
(TIF)

Figure S2 **Evidence of recombination at the target** ***Tsc1*** **gene.** To confirm that recombination had occurred at the *Tsc1* locus in the brains of mice subject to injection of the AAV-Cre, we performed multiplex ligation-dependent probe assay (MLPA). MLPA can be used to determine the extent of recombination of the c to the k (null) allele at *Tsc1* in a quantitative fashion, as described (28,29). Capillary electrophoresis tracings are shown for 5 DNA samples. The peak on the left reflects the abundance of the c allele; that on the right reflects the abundance of the k allele. Samples A–E are: A) control *Tsc1*ck blood DNA sample; B) control *Tsc1^c/c^* mouse brain sample at age 1 month after AAV1-CBA-GFP injection; C) control *Tsc1^c/c^* mouse brain sample at age 3 months after AAV1-CBA-GFP injection; D) *Tsc1^c/c^* mouse brain sample at age 1 month after AAV1-CBA-Cre injection; E) control *Tsc1^c/c^* mouse brain sample at age 3 months after AAV1-CBA-Cre injection. *Note.* The A (control) sample shows roughly equal amounts of signal for the c and k alleles; samples B and C show no k allele signal; and samples D and E show approximately 90% c and 10% k signal.
(TIF)

Figure S3 **MR images from AAV1-CBA-Cre and AAV1-CBA-GFP injected mice.** Coronal (top panels) and sagittal (bottom panels) pseudocolored images are shown for AAV1-CBA-Cre (left panels, two different animals) and AAV1-CBA-GFP (right panels) injected animals. Apparent geographical areas of higher signal abnormally in the cortical/subcortical zones were noted in one AAV1-CBA-Cre injected animal and not in controls.
(TIF)

Table S1 Histological analysis was carried out on *Tsc1^c/c^* mice following P0 ICV injection of AAV1-CBA-Cre or AAV1-CBA-GFP sacrificed at different time points. Severe hydrocephalus was seen in 2 of 10 AAV1-CBA-Cre injected brains, and mild hydrocephalus in 6 of 10 brains in animals sacrificed at 1–5 months of age due to signs of distress, including hunched back, dehydration and weight loss. In contrast, mild hydrocephalus was seen in only 1in 6 brains of animals injected with the AAV1-CBA-GFP control vector.
(XLS)
