# Correction: Stochastic Model of *Tsc1* Lesions in Mouse Brain

**DOI:** 10.1371/annotation/6a5b0a50-27e4-49bc-b82a-9267dd63af53

**Published:** 2013-11-07

**Authors:** Shilpa Prabhakar, June Goto, Xuan Zhang, Miguel Sena-Esteves, Roderick Bronson, Jillian Brockmann, Davide Gianni, Gregory R. Wojtkiewicz, John W. Chen, Anat Stemmer-Rachamimov, David J. Kwiatkowski, Xandra O. Breakefield

The following was incorrectly omitted from the Acknowledgments: "We would like to thank Danielle Morse for the production of AAV-GFP vectors and Dr. Bakhos Tannous Ph.D, Director of Vector Development and Production Core, MGH, Charlestown, MA".

In addition, the name of the third author was spelled incorrectly. The correct name is: Xuan Zhang. The correct citation is: Prabhakar S, Goto J, Zhang X, Sena-Esteves M, Bronson R, et al. (2013) Stochastic Model of *Tsc1* Lesions in Mouse Brain. PLoS ONE 8(5): e64224. doi:10.1371/journal.pone.0064224 

