# Supplementary figures and images for: Stochastic Model of Tsc1 Lesions in Mouse Brain
Source: PLoS One. 2013 May 16;8(5):e64224. doi: 10.1371/journal.pone.0064224 (PMC3655945; doi:10.1371/journal.pone.0064224)

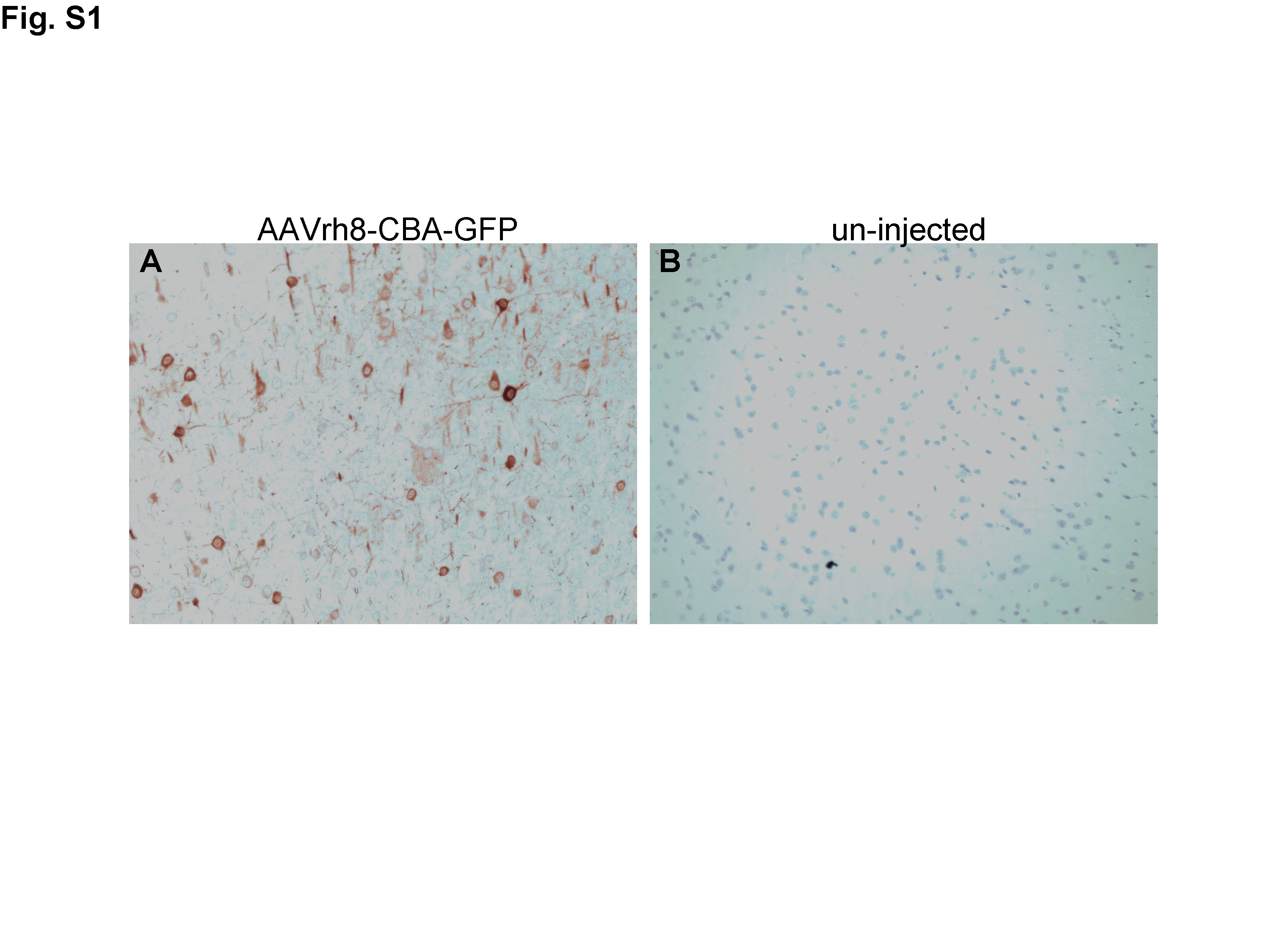

Supplement: Figure S1 — GFP staining on the AAVrh8-CBA-GFP injected brains. Brains of AAVrh8-CBA-GFP injected at P0 and uninjected Tsc1c/c mice at P30 at 110 days were stained for GFP and counter stained with haematoxylin. Positive staining was revealed throughout the brain, shown here in the cortex in the cortex and the ventricles in the AAV injected mice. Magnification = 20X. (TIF) [file pone.0064224.s001.tif]

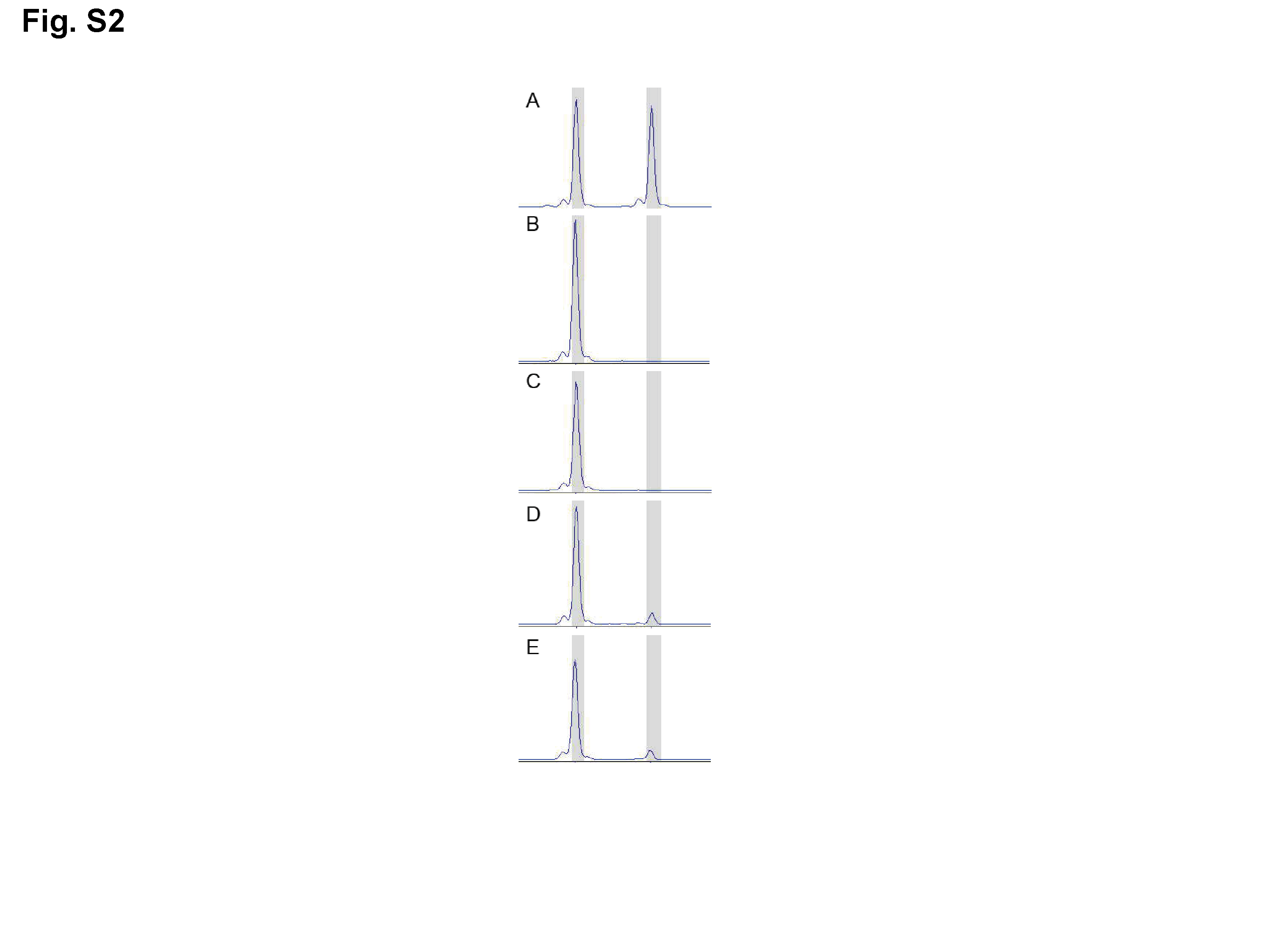

Supplement: Figure S2 — Evidence of recombination at the target Tsc1 gene. To confirm that recombination had occurred at the Tsc1 locus in the brains of mice subject to injection of the AAV-Cre, we performed multiplex ligation-dependent probe assay (MLPA). MLPA can be used to determine the extent of recombination of the c to the k (null) allele at Tsc1 in a quantitative fashion, as described (28,29). Capillary electrophoresis tracings are shown for 5 DNA samples. The peak on the left reflects the abundance of the c allele; that on the right reflects the abundance of the k allele. Samples A–E are: A) control Tsc1ck blood DNA sample; B) control Tsc1c/c mouse brain sample at age 1 month after AAV1-CBA-GFP injection; C) control Tsc1c/c mouse brain sample at age 3 months after AAV1-CBA-GFP injection; D) Tsc1c/c mouse brain sample at age 1 month after AAV1-CBA-Cre injection; E) control Tsc1c/c mouse brain sample at age 3 months after AAV1-CBA-Cre injection. Note. The A (control) sample shows roughly equal amounts of signal for the c and k alleles; samples B and C show no k allele signal; and samples D and E show approximately 90% c and 10% k signal. (TIF) [file pone.0064224.s002.tif]

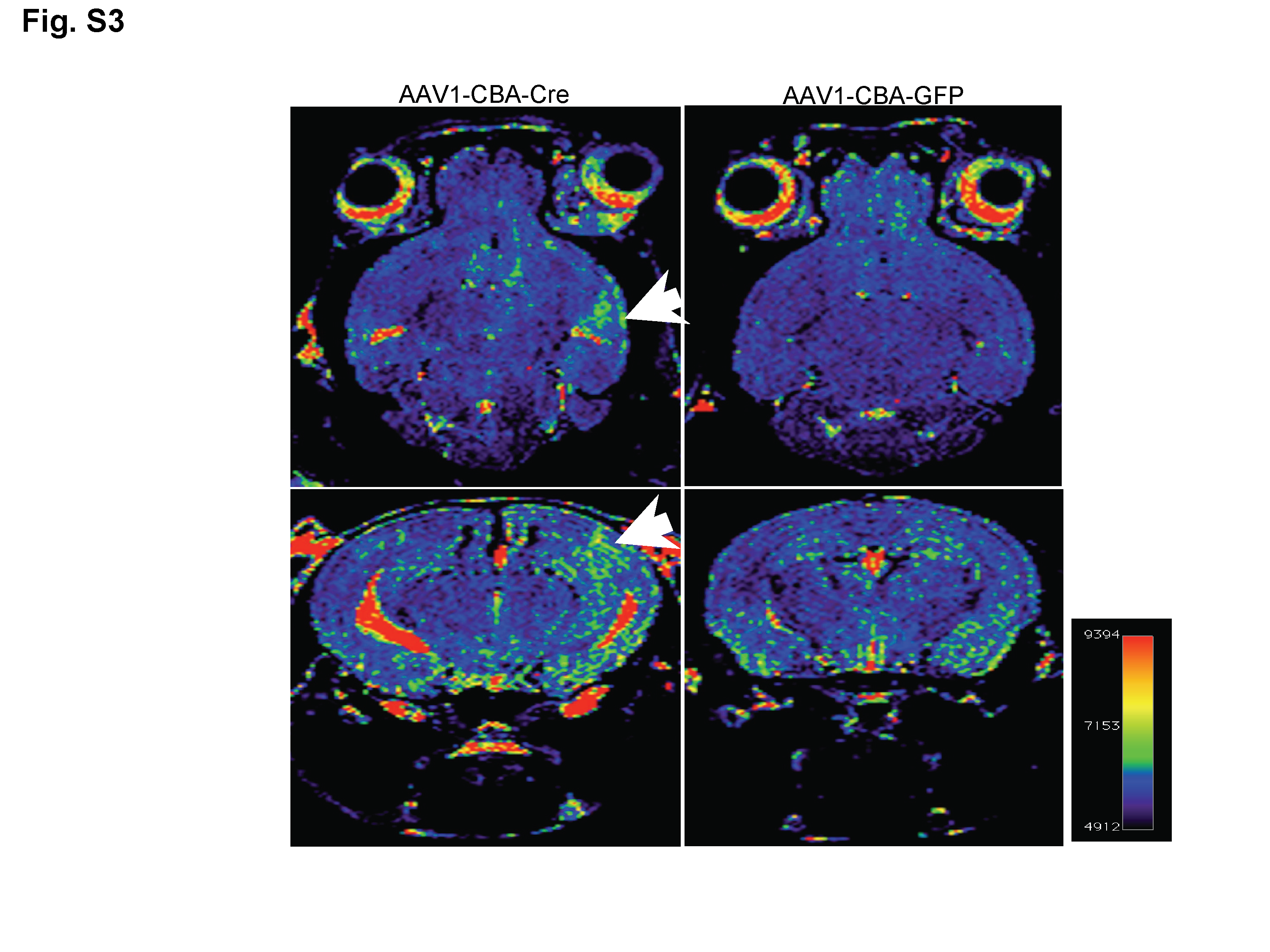

Supplement: Figure S3 — MR images from AAV1-CBA-Cre and AAV1-CBA-GFP injected mice. Coronal (top panels) and sagittal (bottom panels) pseudocolored images are shown for AAV1-CBA-Cre (left panels, two different animals) and AAV1-CBA-GFP (right panels) injected animals. Apparent geographical areas of higher signal abnormally in the cortical/subcortical zones were noted in one AAV1-CBA-Cre injected animal and not in controls. (TIF) [file pone.0064224.s003.tif]
